# Complete Genome Sequence of Strain BB001, a Novel Epibiont Bacterium from the Candidate Phylum *Saccharibacteria* (TM7)

**DOI:** 10.1128/MRA.00810-20

**Published:** 2020-08-20

**Authors:** Eleanor I. Lamont, Erik L. Hendrickson, Jeffrey S. McLean, Xuesong He, Batbileg Bor

**Affiliations:** a Department of Periodontics, University of Washington, Seattle, Washington, USA; b Department of Microbiology, The Forsyth Institute, Cambridge, Massachusetts, USA; c Department of Oral Medicine, Infection and Immunity, Harvard School of Dental Medicine, Boston, Massachusetts, USA; University of Rochester School of Medicine and Dentistry

## Abstract

Strain BB001 is cultivated from the human oral cavity on its basibiont bacterial host *Actinomyces* sp. It is an ultrasmall bacterium with a reduced genome that grows obligately on its bacterial host. BB001 is the first member of human microbiome taxon 957.

## ANNOUNCEMENT

*Saccharibacteria* (TM7) are members of the candidate phylum radiation (CPR) group (1, 2). CPR organisms comprise >25% of all bacterial diversity and have an ultrasmall cell size and reduced genome (1, 3). The first member of the *Saccharibacteria*, Nanosynbacter lyticus strain TM7x (GenBank accession number GCA_000803625.1), was cultivated from the human oral cavity (4–7). Consequently, additional *Saccharibacteria* strains were cultivated on their bacterial hosts (8, 9). Strain BB001 was isolated on its bacterial host *Actinomyces* sp. strain F0337 from the healthy human oral cavity, as previously described (8). The saliva was filtered through a 0.45-μm filter to separate ultrasmall bacteria. The filtrate was mixed with candidate bacterial hosts (e.g., *Actinomyces*, *Corynebacterium*, *Pseudopropionibacterium*, and *Leptotrichia* spp.) to isolate *Saccharibacteria* species. *Saccharibacteria* that preferred the host grew in coculture. Ultrasmall bacteria that did not grow were cleaned by sequential passaging and agar plating. A detailed procedure can be found in reference 8. BB001 is the first isolate of human microbiome taxon 957 (HMT-957).

For genomic DNA isolation, BB001 was cocultured with F0337 in 400 ml of brain heart infusion medium under microaerobic conditions. The coculture was filtered through a 0.45-μm filter to remove hosts. The filtrate containing BB001 was then pelleted by centrifugation. Genomic DNA was extracted using the Epicentre MasterPure kit (Lucigen). Library preparation and sequencing were completed at Johns Hopkins Deep Sequencing and Microarray Core. Genomic DNA was sheared to 10- to 20-kb sizes using a Covaris g-TUBE and was purified by AMPure-XP beads (Agencourt Bioscience). Size selection and cleanup were performed using BluePippin (Sage Science). The library was sequenced on a PacBio RS II instrument with P6/C4 chemistry on one single-molecule real-time (SMRT) cell per library. Default parameters were used for all software. The reads were assembled using the SMRT Analysis software 2.3.0 HGAP4 (10) with the standard protocol for adaptor trimming, quality filtering, and error correction and annotated with the NCBI Prokaryotic Genome Annotation Pipeline using the best-placed reference protein set (GeneMarkS-2) (8, 11, 12). Methylation motifs were detected using the SMRT Analysis software version 2.3.0 Base_Modification_and_Motif_Analysis pipeline (10).

There were 50,502 raw reads covering 580,618,123 bases. The mean read length was 11,496 bases, and the *N*_50_ read length was 17,758 bases. The genome was assembled into a single contig 825,455 bp long and was circularized to 782,344 bp by removing 43,111 bp at each end. The average reference coverage was 620×. It had a G+C content of 47.9%. Gene annotation identified a total of 809 genes with 49 RNAs. A neighbor-joining 16S rRNA phylogenetic tree (8) revealed that BB001 is closely associated with TM7x but is in a distinct clade designated HMT-957. The tree was created by aligning full-length 16S rRNA sequences of reference *Saccharibacteria* sequences using MEGA X (13). Broader TM7 phylogeny can be found in references 14 and 15. BB001 had 99.4% and 73.1% similarities to TM7x by 16S rRNA and whole-genome amino acid identity comparisons, respectively. Roary was used to determine similarity and amino acid identity (16). BB001 is a novel cultured member of *Saccharibacteria* and is given the provisional name Nanosynbacter featherlites strain BB001 HMT-957.

### Data availability.

This genome has been deposited in GenBank under the accession number CP040004.1 and SRA accession number SRR11847359.
